# Comparing CMIP-3 and CMIP-5 climate projections on flooding estimation of Devils Lake of North Dakota, USA

**DOI:** 10.7717/peerj.4711

**Published:** 2018-04-30

**Authors:** Gehendra Kharel, Andrei Kirilenko

**Affiliations:** 1 Department of Natural Resource Ecology and Management, Oklahoma State University, Stillwater, OK, USA; 2 Department of Tourism, Recreation & Sport Management, University of Florida, Gainesville, FL, USA

**Keywords:** Devils lake, SWAT, Climate change, CMIP-5, CMIP-3, General circulation models, Red river basin of the North

## Abstract

**Background:**

Water level fluctuations in endorheic lakes are highly susceptible to even slight changes in climate and land use. Devils Lake (DL) in North Dakota, USA is an endorheic system that has undergone multi-decade flooding driven by changes in regional climate. Flooding mitigation strategies have centered on the release of lake water to a nearby river system through artificial outlets, resulting in legal challenges and environmental concerns related to water quality, downstream flooding, species migration, stakeholder opposition, and transboundary water conflicts between the US and Canada. Despite these drawbacks, running outlets would result in low overspill risks in the next 30 years.

**Methods:**

In this study we evaluated the efficacy of this outlet-based mitigation strategy under scenarios based on the latest IPCC future climate projections. We used the Coupled Model Intercomparison Project CMIP-5 weather patterns from 17 general circulation models (GCMs) obtained under four representative concentration pathways (RCP) scenarios and downscaled to the DL region. Then, we simulated the changes in lake water levels using the soil and water assessment tool based hydrological model of the watershed. We estimated the probability of future flood risks under those scenarios and compared those with previously estimated overspill risks under the CMIP-3 climate.

**Results:**

The CMIP-5 ensemble projected a mean annual temperature of 5.78 °C and mean daily precipitation of 1.42 mm/day; both are higher than the existing CMIP-3 future estimates of 4.98 °C and 1.40 mm/day, respectively. The increased precipitation and higher temperature resulted in a significant increase of DL’s overspill risks: 24.4–47.1% without release from outlets and 3.5–14.4% even if the outlets are operated at their combined full 17 m^3^/s capacity.

**Discussion:**

The modeled increases in overspill risks indicate a greater frequency of water releases through the artificial outlets. Future risk mitigation management should include providing a flood warning signal to local resource managers, and tasking policy makers to identify additional solution measures such as land use management in the upper watershed to mitigate DL’s flooding.

## Introduction

Impacts of climate change on both natural and human systems at local, regional and global scales are continuously being observed, assessed, reported, and documented. Impact studies utilize data related to historical observation together with scenarios of future changes in climate to project related changes in environment, economics, and society and to evaluate possible response strategies. One of the most concerning potential impacts of climate change is elevated frequency and/or intensity of flooding. The risks of “great floods” (the floods with discharges exceeding 100-year level) are projected to increase with the flood rate increasing two to eight times as compared to historical comparisons (Milly et al., 2002). In fact, frequency of flooding has increased in 42% and decreased in 18% of global land area (Hirabayashi et al., 2013). Yet, as the Intergovernmental Panel on Climate Change (IPCC) report on extreme events notes, the confidence in projections of these changes is low “due to limited evidence and because the causes of regional changes are complex” (IPCC, 2012, p. 113). Three sources of uncertainties about future climate projections include the differences between models in the parameterization of physical processes, the uncertainty in future driving forces of climate, and the internal variability of climate which can lead to low confidence in projected extreme events (Hawkins & Sutton, 2011; Knutti & Sedláček, 2013; Sillmann et al., 2013). Endorheic lakes are water bodies with a catchment area but without an outflow, and thus represent a relatively simple hydrologic system. In this respect, endorheic lakes provide a good testbed for examining flood related risks of climate change.

**Figure 1:**
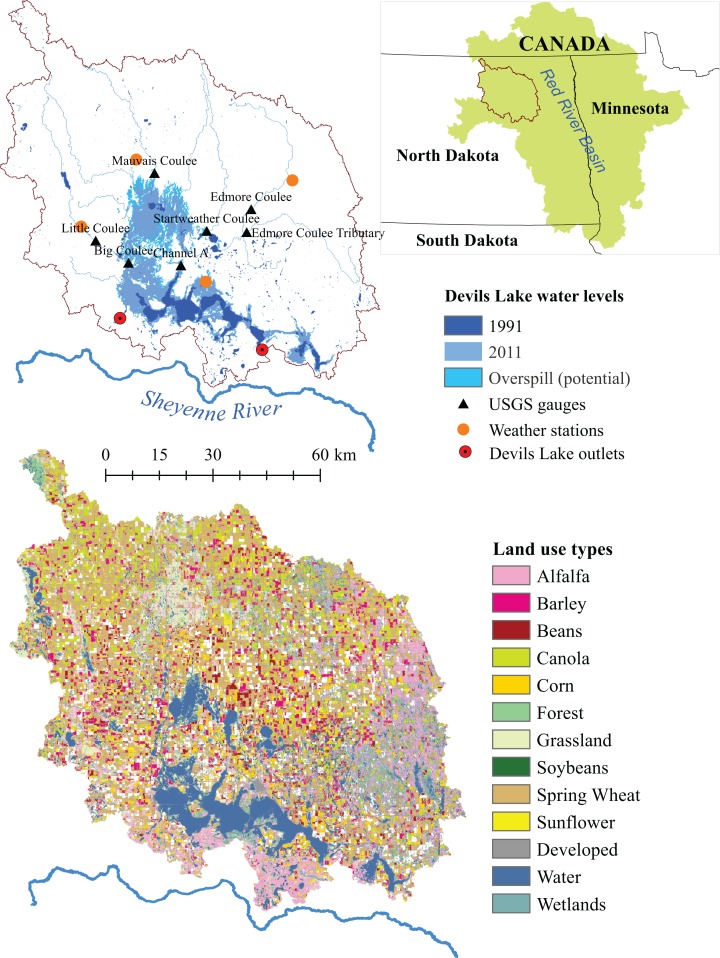
Devils Lake watershed located in Northern Great Plains area close to the USA-Canada border within the Souris-Red-Rainy drainage region. Geospatial data were obtained from the North Dakota GIS Hub Data Portal (https://gishubdata.nd.gov/) and the USDA-National Agricultural Statistics Cropland Data Layer (2008).

The amplification effect evident in endorheic watersheds makes them important for detection of regional changes in climate, because even relatively small changes in local temperature and precipitation extended over years leads to highly visible changes in water level and lake area, imposing a direct impact on the livelihood of lake-dependent populations. Kharel & Kirilenko (2015) provided numerous examples of closed lakes across the globe with receding (e.g., Aral Sea, Lake Chad, and Qinghai Lake) or rising (e.g., Caspian Sea and Mar Chiquita) waters related to changing climate and land use (see also Rosenzweig et al., 2007). While endorheic lakes most commonly experience receding water level due to human activities, DL, located in a sparsely populated Northern Great Plains area close to the US—Canada border (United States Geological Survey (USGS) Souris-Red-Rainy drainage region, see Fig. 1), has experienced frequent flooding problems.

Since its formation at the end of the Wisconsin glacial period roughly 10,000 years ago, DL has experienced multiple water level fluctuations between dominantly dry (6,500 years ago) and at least two overspills beyond the watershed boundary between 800 and 1,200 years ago (Murphy, Fritz & Fleming, 1997). The latest episode of long-term retreat of DL’s water level was traced from 1867 when it was first measured by the USGS at 438 m above mean sea level (amsl) until the 1940s when it decreased to 427 m amsl. The current long-term trend of DL’s water level increasing started in 1940 and accelerated in the late 1980s following regional climate shifts towards higher precipitation in May, June, and early fall season (Johnson et al., 2005; Vecchia, 2002). During this period the DL’s water level increased by 10 m with the highest level of 443.27 m amsl recorded in June of 2011. DL directly responds to regional climate variability, which results in large fluctuations of its water level. The impacts of DL’s water level change were highly visible and costly. Between 1991 and 2011, DL’s water level rose by 10 m inundating more than 650 km^2^ of prime farm lands, and other infrastructure including roads and bridges resulting in nearly 1.5 billion US dollars in damage and mitigation efforts (Devils Lake Basin Joint Water Resource Board, 2013; Noone, 2012).

In 2011, the lake was only 1.1 m below its natural overspill (444.4 m) to the nearby Sheyenne River, a tributary of the Red River of the North that connects to Lake Winnipeg in Canada, which in turn is a part of the Hudson Bay watershed. In the event of overspill, down-cutting through the outlet could allow more than 40% of the stored water to empty into the Sheyenne River at a rate of 340–453 m^3^/s (Larson, 2012), leading to devastating downstream flooding of many communities, water quality degradation, and potential biota transfer to the downstream water resources in the Red River basin (US Army Corps of Engineers, 2003). The potential for downstream flooding and water quality issues associated with draining of DL’s salty water are matters of serious concern to downstream stakeholders including communities in North Dakota and Minnesota (USA), and Manitoba (Canada). One of the management efforts to mitigate major flooding included the construction and operation of two different outlets with the combined maximum pumping capacity of 17 m^3^/s.

Uncertainty about future lake levels has contributed to the low effectiveness and high costs of DL flood mitigation, which in turn has led to severely discounted long-term risks (Zheng, Barta & Zhang, 2014). Pioneering research by Vecchia (2002, 2008, 2011) estimated the probability of future DL overspill under the assumption of a prolonged wet phase of the regional climate. Those studies however did not consider the impacts of global climate change on regional hydrology. Climate change impacts on DL’s flooding were taken into account by Kharel & Kirilenko (2015), who estimated the probability of future DL levels and associated uncertainty by employing an ensemble of climate change projections used in the 2007 report of the IPCC (Solomon et al., 2007). Further, Kharel, Zheng & Kirilenko (2016) estimated the role of land use change within the DL watershed in the observed flooding.

In this paper, we used the 2013 CMIP-5 IPCC climate projections (Stocker et al., 2013) to update the earlier projections of DL flood risks. The main objective of this study is to evaluate the effectiveness of outlet-based flood mitigation strategy in DL under the projected changes in CMIP5 precipitation and temperature and compare those new results to the earlier CMIP-3 projections. This work will provide better scientific support for management decisions by taking the latest climate change science into consideration.

### Regional climate change

The primary approach to estimating future climate-related risks employs synthetic weather patterns obtained from GCM simulations under various scenarios of future changes in radiative forcing related to human activities. However, two potential problems limit the effectiveness of using the GCM projections as an input to a hydrological model. The first is a well-known problem of systematic bias in GCM projections, exacerbated by a mismatch between their coarse scale and the fine scale required for a watershed-level study. The second problem is accounting for the uncertainty that arises from differences between GCMs. Multiple downscaling methods have been developed and tested to correct the bias and provide a link between the scales of GCMs and required input of hydrological models (Chen et al., 2012; Maraun, 2016). Wood et al. (2004) provided a comparison of the hydrological simulations obtained with different downscaling methods.

The model-related uncertainty within the second problem results from different projections of future patterns of climate parameters obtained with different GCMs. While the majority of hydrological studies employ the output of a single GCM, some researchers have used multiple GCMs to account for model-related uncertainty. For example, Hirabayashi et al. (2013) employed the output from 11 GCMs in a study of modifications in global flood risks to estimate future changes to flood return frequencies in 29 river basins worldwide, together with the uncertainty of those estimates. Hydrological modeling with a multi-model, multi-run ensemble of GCM climate projections helps to reduce future climate uncertainty in the decision-making process (Brown et al., 2012).

The World Climate Research Program (WCRP) designated the Coupled Model Intercomparison Project (CMIP) to store and distribute the output of participating GCMs obtained under a standardized set of scenarios. The majority of existing flood risk studies use the GCM projections obtained from the CMIP-3 project, which was released for the Working Group I contribution to the IPCC’s Fourth Report (Solomon et al., 2007). Meanwhile, an updated ensemble of GCM projections, labeled CMIP-5, has been prepared for the fifth IPCC report (Stocker et al., 2013). CMIP-5 models have a higher spatial resolution and improved physical representation and integration of the processes in the atmosphere, ocean, and land (Flato et al., 2013; Knutti & Sedláček, 2013). One of the major advancements in CMIP-5 over CMIP-3 is the inclusion of the effects of land use changes associated with agriculture, urbanization and deforestation that impacts climate through the alteration of albedo, aerodynamic roughness, and water-holding capacity (Flato et al., 2013), which makes them more relevant for the decision-making process. However, uncertainties related to aerosol-cloud dynamics (Stanfield et al., 2014), and a lack of improvement in capturing the North American monsoon system and North Atlantic subtropical high as compared to the CMIP-3 models (Geil, Serra & Zeng, 2013; Wuebbles et al., 2014) existed in CMIP-5 GCMs. Wuebbles et al. (2014) analyzed CMIP-5 models and found no significant changes in the overall magnitude of projected temperature and precipitation over the continental US as compared to the CMIP-3 projections.

Additionally, the CMIP-5 projections represent an improvement over CMIP-3 because they were obtained under less proscriptive sets of anthropogenic climate forcing scenarios. The scenarios used in CMIP-3 projections were represented by alternative story lines describing future changes in human population, energy consumption, use of fossil fuels and other modifications in society and economics, as specified in Nakicenovic et al. (2000). The approach used in CMIP-5 employed a set of four RCP scenarios that describe alternative paths to reach specified radiative forcing of climate change so that different socioeconomic changes may lead to the same level of radiative forcing (van Vuuren et al., 2011). The four RCP scenarios include “mitigation” (RCP 2.6) with low radiative forcing, “stabilization” (RCPs 4.5 and 6.0), and “very high emission” (RCP 8.5) (Flato et al., 2013) with the index specifying the related radiative forcing (W/m^2^) by year 2100 (Meinshausen et al., 2011).

Radical modification of climate change scenarios together with advancement in GCM projections inspired multiple comparison studies of hydrological projections made with CMIP-5 and CMIP-3 GCM ensembles. A large assessment of climate projections for the US territory (Wuebbles et al., 2014) found an improvement in replication of the seasonal cycle of precipitation in the CMIP-5 ensembles. In respect to future changes in heavy precipitation, CMIP-3 and CMIP-5 agreed on an increase of the number of incidents, but the authors found large differences between the ensembles in the rate of heavy precipitation increase. Extreme precipitation events are estimated to increase by 5–25% in the late century (2081–2100) as compared to historically (1986–2015) in the DL region along the North Dakota–Minnesota border (Wuebbles et al., 2014). Mahat, Ramírez & Brown (2017) studied the implications of CMIP-5 projections on snow and water yield in the contiguous US and found an increase in average annual temperature (+4%) and precipitation (+3%) led to decreased water yield (−4%) compared to the historical climate. The differences between ensembles have led to re-estimation of the flood risks in many regions; for example, Ficklin et al. (2016) compared snow-dependent hydrologic projections in the western US and found that, while in many basins the projections with both ensembles agreed, the CMIP-5 projections demonstrated increased streamflow in some key regions such as the Colorado River basin. In the Chicago area, CMIP-5 projections indicated up to 9% increase in frequency of 100-year precipitation events as compared to the CMIP-3 projections (Markus et al., 2018). In British Columbia, Canada, CMIP-5 projections indicated warmer and wetter conditions than CMIP-3 resulting in increased winter and spring streamflow (Schnorbus & Cannon, 2014). In contrast, Mahat, Ramírez & Brown (2017) found the largest increase in temperature (+3.4% to +6.0%) and precipitation (+3%) under the CMIP-5 climate in eastern North Dakota and western Minnesota, leading to decreased water yield (−6% or more) in the region.

## Methods and Data

The Soil and Water Assessment Tool (SWAT) was calibrated and validated for the DL watershed using historical data for the period 1991 to 2010. Then, two sets of downscaled CMIP-3 and CMIP-5 climate scenarios were developed for the same DL region. Finally, future flood risk projections were obtained and compared for both sets, as described below.

### Hydrological model

The hydrological model of the DL watershed was developed using the SWAT modeling framework (Arnold et al., 1998). The DL watershed was delineated using a 10 m resolution digital elevation model (DEM) (http://ned.usgs.gov/downloads.html). A stream network extracted from the USGS National Hydrography Dataset was burned-in to the DEM to divide the watershed into 12 sub-basins with the associated topographic characteristics. A crop layer (USDA-National Agricultural Statistics Cropland Data Layer, 2008) was used to derive land use coverage for each sub-basin. The watershed is comprised of 13 different land uses with agriculture (spring wheat, soybeans, corn, canola, barley, beans, and alfalfa) occupying nearly 65% of the watershed (Fig. 1). Soil information in the watershed was obtained from the soil survey geographic database—SSURGO (Soil Survey Staff, Natural Resources Conservation Service, United States Department of Agriculture, 2018). The watershed was divided into two slope classes: 0–5% and greater than 5%. Then these land use types, and soil and slope classes, were overlaid to produce 156 unique hydrologic response units (HRU). Historical climatic condition in the watershed was represented by daily observations of precipitation and temperature data for four stations inside the watershed for 1981 to 2010 obtained from the USDA Agricultural Research Service database (http://ars.usda.gov). The model simulated water flow in each HRU, which was then integrated at the sub-basin and the entire watershed levels. DL’s water level change was estimated from the balance of lake inflows, lake evaporation, and water outflow from the lake’s artificial outlets.

### Model calibration and validation

**Figure 2:**
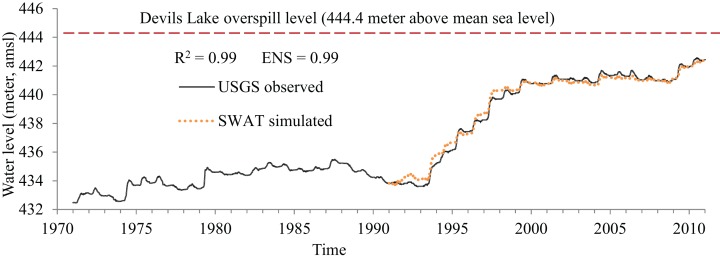
Devils Lake water level fluctuations: SWAT-simulated compared with the observations. SWAT simulations start from 1991 to 2010 as indicated in a dotted line. Red dashed line indicates the lake’s overspill level at 444.4 m from the above mean sea level (amsl).

Model performance was evaluated by comparing the SWAT simulated daily streamflow with the observations from the seven USGS stream gage stations within the watershed for the period of 1991–2010. Since not all the stations had continuous data available for the entire time period, model calibration and validation were carried for different time intervals for different stations. The 1991–2010 period was selected for model calibration and validation to capture the nonlinear dynamics of DL during this time period when the lake level rose dramatically. The automatic calibrator SWAT-CUP (Abbaspour, 2011) was used for model calibration, validation, and sensitivity analysis. The Latin hypercube parameter sampling method (McKay, Beckman & Conover, 2000) in SWAT-CUP was used to identify the parameters that were most sensitive to the model. We found that the parameters related to snow (SFTMT, SMTMP, SMFMX, SMFMN, TIMP, SNOCOVMX), evapotranspiration (ET) (ESCO, EPCO, EVRSV), water routing (CH_N2, CH_N1, ALPHA_BNK), surface runoff (CN_2_, SURLAG), and groundwater (ALPHA_BF) were the most sensitive (Table 1). These 15 parameters were scrutinized to calibrate the model. The calibrated values of these parameters in each sub-basin are listed in Table S1. Additionally, the values of surface runoff curve numbers (CN_2_) for all 13 land uses in the watershed are listed in Table S2. Two statistical matrices—the coefficient of determination *R*^2^ and Nash–Sutcliffe model efficiency (ENS) were used to evaluate the overall performance of the model. Both *R*^2^ and ENS put an emphasis on high flow, which is important for our simulation goals. The values of *R*^2^ > 0.5 are considered satisfactory, *R*^2^ > 0.6 good, and *R*^2^ > 0.7 very good (Moriasi et al., 2007). For evaluating ENS, values greater than 0.5 are considered satisfactory, those greater than 0.65 are good, and those greater than 0.75 are very good (Moriasi et al., 2007). Based on these criteria, the comparison of the simulated vs. observed daily streamflow indicated acceptable model performance (customary agreed as ENS > 0.5): *R*^2^ ∼ 0.57–0.88 and ENS ∼ 0.51–0.86, with the range representing different USGS streamflow stations (Table 2; Fig. S1). Additionally, the model’s skill in simulating DL’s water level was estimated by comparing SWAT simulated and observed 1991–2010 DL water levels and was found highly correlated (*R*^2^ = 0.99; ENS = 0.99) (Fig. 2).

**Table 1:** Model calibration parameters and associated values.

SWAT parameters	Description	Calibrated values/ranges	Acceptable range
SFTMT	Snowfall temperature (°C)	0.58	−5 to 5
SMTMP	Snowmelt temperature (°C)	1.28	−5 to 5
SMFMX	Snowmelt factor on June 21 (mm/H_2_O/°C-day)	5.5	0–10
SMFMN	Snowmelt factor on December 21 (mm/H_2_O/°C-day)	2.25	0–10
TIMP	Snowpack temperature lag factor (unit less)	0.33	0–1
SNOCOVMX	Snowpack water content at 100% coverage (mmH_2_O)	14	1–20
CN_2_	Runoff curve number for soil moisture condition 2 (unit less)	Varies between the land uses; ∼ −7% of default values**	±25% of default values, varies between the land uses
SURLAG	Surface runoff lag coefficient (unit less)	0.1–0.9*	0–24
ALPHA_BF	Baseflow recession constant (days)	0.37	0–1
ESCO	Soil evaporation compensation factor (unit less)	0.02–0.8*	0–1
EPCO	Plant evaporation compensation factor (unit less)	0.1–1.0*	0–1
CH_N_2_	Main channel manning’s *N* (unit less)	0.02–0.1*	0.01–0.15
ALPHA_BNK	Bank storage recession constant (days)	0.1–0.88*	0–1
CH_N_1_	Tributary channel manning’s *N* (unit less)	0.014–0.08*	0.01–0.15
EVRSV	Lake evaporation coefficient (unit less)	0.78	0–1

**Notes:**

* See Table S1 for parameter values in each of the 12 sub-basins.

** See Table S2 for CN_2_ values for all 13 land use types in each of the 12 sub-basins.

**Table 2:** Estimation of SWAT model performance.

USGS gauging stations	Model performance			
	Calibration		Validation	
	*R*^2^	ENS	*R*^2^	ENS
Big coulee* (#05056400)	0.65	0.61	0.70	0.66
Channel A* (#05056407)	0.88	0.86	0.77	0.70
Edmore coulee** (#05056200)	0.72	0.71	0.75	0.74
Edmore coulee Tributary** (#05056215)	0.76	0.74	0.69	0.69
Little coulee*** (#05056340)	0.75	0.73	0.71	0.56
Mauvais coulee** (#05056100)	0.57	0.55	0.63	0.63
Starkweather coulee** (#05056239)	0.65	0.61	0.70	0.66

**Notes:**

* Calibration (1991–1995) and validation (1996–1998).

** Calibration (1991–1998) and validation (2001–2010).

*** Calibration (1998–2004) and validation (2005–2010).

### Climate projections

Future climate projections were generated from 15 CMIP-3 and 17 CMIP-5 GCMs for two time periods representative of the 2020s and 2050s climates, as follows. The CMIP-3 weather pattern ensemble used integrations of the following GCMs: BCC-CSM1.1, BCC-CSM1.1-m, CSIRO-Mk3.6.0, FIO-ESM, GFDL-CM3, GFDL-ESM2G, GFDL-ESM2M, GISS-E2-R, HadGEM2-ES, IPSL-CM5A-MR, MIROC5, MIROC-ESM, MIROC-ESM-CHEM, MRI-CGCM3, and NorESM1-M. For each model, we used climate parameters obtained from model simulations under three emission scenarios from the fourth IPCC assessment: A1B, A2, and B1 (35 model-scenario combinations as some of the GCMs lack all three scenarios). In a similar way, the CMIP-5 ensemble was developed using integrations of the following GCMs: BCC-CSM1.1, BCC-CSM1.1(m), CSIRO-Mk3.6.0, FIO-ESM, GFDL-CM3, GFDL-ESM2G, GFDL-ESM2M, GISS-E2-H, GISS-E2-R, HadGEM2-ES, IPSL-CM5A-LR, IPSL-CM5A-MR, MIROC-ESM, MIROC-ESM-CHEM, MIROC5, MRI-CGCM3, NorESM1-M. We used four emission scenarios from the fifth IPCC assessment: RCP 2.6, RCP 4.5, RCP 6.0, and RCP 8.5 (68 model-scenario combinations).

### Downscaled climate

To correct GCM projections from the spatial resolution bias described above, we applied two stochastic weather generators; LARS-WG (Semenov et al., 1998) and MarkSim (Jones & Thornton, 2013). These weather generators use the observed weather together with a GCM output to produce daily synthetic weather patterns representative of historical or future climates. Both LARS-WG and MarkSim have been utilized to downscale climate forecasts in studies worldwide; for example, MarkSim is included as a part of the decision support system for agrotechnology transfer family of crop yield models. Schematically, these weather generators are similar. First, wet–dry day series are modeled using a Markov Chain (MarkSim) or a semi-empirical distribution (LARS-WG). Then, precipitation is distributed over the wet days. Finally, daily values of other weather variables are generated conditioned on precipitation (Khazaei et al., 2013). Selection of LARS-WG for the CMIP-3 study was based on its better performance at simulating daily precipitation amounts (Chen & Brissette, 2014), possibly related to its semi-empirical distribution-based simulation of precipitation vs. the parametric distribution-based scheme frequently used in weather generators. MarkSim was used for the CMIP-5 ensemble, because LARS-WG had not been adapted to the CMIP-5 at the time of this research.

### Replication of historical climate

The skill of LARS-WG and MarkSim weather generators applied to CMIP-3 and CMIP-5 simulations was assessed by comparing the synthetic temperature and precipitation against the NOAA “current climate” 1981–2010 temperature and precipitation observations. Monthly precipitation had a moderate bias (max = 17% for LARS-WG data; max = 21% for MarkSim data), which was judged to be acceptable given spatial precipitation variability. At the same time, mean monthly biases over the warm period and over the cold period were small (lesser than 5%) and the annual precipitation bias was less than 1% for both datasets. For temperature, monthly synthetic minimum and maximum temperatures had a moderate bias up to 1.6 °C with negligible bias (below 0.3 °C) for the annual mean of minimum and maximum daily temperatures. Overall, LARS-WG and MarkSim both demonstrated acceptable performance.

### Flood mitigation scenarios

DL flood management for the past decade has heavily relied on the two artificial outlets which pump DL water to the Sheyenne River. These outlets have a combined pumping capacity of 17.0 m^3^/s, equivalent to a maximum of 0.36 km^3^ of water annually. This annual maximum is unlikely to be reached because these outlets are usually operational only from May to October given the winter weather freezing condition. Also, the current regulatory constraints on water quality and quantity in the Sheyenne River prevent the operation of these outlets at full-capacity. Kharel & Kirilenko (2015) used an ensemble of 15 IPCC CMIP-3 GCMs in a SWAT hydrologic model to evaluate DL overspill risks, and, showed that running the full-capacity outlet from May to October under forecast climate conditions led to negligible risk of the lake’s overspill, and Kharel, Zheng & Kirilenko (2016), using the same CMIP-3 climate ensemble, found a 16% overspill risk if outlets are not utilized. Kharel, Zheng & Kirilenko (2016) also showed that increasing alfalfa production from 7.4% of land to 18.5% on agricultural areas would lower the overspill probability (OP) to 7.4%, demonstrating the hydrological importance of land use change as a flood risk management tool. In this study, we used similar scenarios: employment of full-capacity outlet, increasing alfalfa land cover to 18.5%, and their combination (Table 3) to estimate the flood mitigating capacity of these flood management strategies under the CMIP-5 climate.

**Table 3:** Flood mitigation scenarios.

Flood mitigation scenario	Description	Source
Full-capacity outlet	Outlets at 17.0 m^3^/s water pumping operation from May to October	Kharel & Kirilenko (2015)
Incentivized alfalfa	No outlet operation; alfalfa land cover in the watershed increase from 8.3% to 18.5% of the watershed	Kharel, Zheng & Kirilenko (2016)
Full-capacity outlet + incentivized alfalfa	Outlets at 17.0 m^3^/s from May to October; 18.5% of watershed under alfalfa production	Kharel & Kirilenko (2015); Kharel, Zheng & Kirilenko (2016)

## Results

### Climate change projections

**Figure 3:**
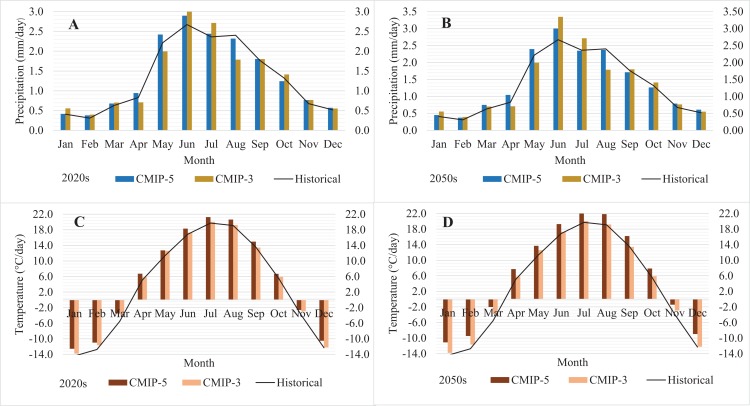
Average monthly precipitation (A, B) and temperature (C, D) under the historical (1981–2010), CMIP-3 and CMIP-5 (2020s and 2050s) climate.

In the DL watershed, both the CMIP-3 and the CMIP-5 ensemble climate projections showed an increase in average temperature and precipitation compared to the current climate (1981–2010); for detail see Table 4 and Fig. 3. The CMIP-5 ensemble showed an overall increase in mean annual temperature (1.71 °C in 2020s and 2.96 °C in 2050s) and increase in mean daily precipitation (3.9% in 2020s and 5.5% in 2050s, respectively) compared to the 1981–2010 historical average. All CMIP-5 GCMs projected a substantial increase in the annual average temperature by 0.61–6.3 °C compared to the historical average. However, we found a significant variation between the scenarios and GCM outputs for precipitation, with the range of −10.4 to +21.4%.

**Table 4:** The 2020s and 2050s climate projections downscaled from CMIP-5 and CMIP-3 ensembles.

Climate	CMIP-5			CMIP-3		
	Scenario	Temp °C	Pcp %	Scenario	Temp °C	Pcp %
2020s	RCP2.6	1.72	3.7	–		
	RCP4.5	1.74	4.4	B1	0.72	2.2
	RCP6.0	1.53	2.9	A1B	0.84	2.2
	RCP8.5	1.84	4.4	A2	0.78	3.7
2050s	RCP2.6	2.30	4.4	–		
	RCP4.5	3.02	6.6	B1	1.85	3.7
	RCP6.0	2.68	3.7	A1B	2.56	4.4
	RCP8.5	3.84	7.4	A2	2.42	2.2

**Note:**

The change of the multi-year average annual temperature (Temp, °C) and annual precipitation (Pcp, percentage), as compared to the 1981–2010 climate. The CMIP-5 and CMIP-3 scenarios are arranged in the order of their approximate correspondence.

Notably the CMIP-5 ensemble generally projected increases in both temperature and precipitation, as compared to the CMIP-3 ensemble. Under the CMIP-5 ensemble, the average annual temperature was 5.78 °C compared to 4.98 °C for CMIP-3, and the average daily precipitation was 1.42 mm/day compared to 1.40 mm/day for CMIP-3 for 2020s and 2050s. Further, CMIP-5 integrations projected even higher precipitation change in April (+6%) and May (+13%) compared to the CMIP-3 projections, the seasonal change responsible for majority of the DL water level increase.

### Devils Lake watershed hydrology and overspill risks

**Figure 4:**
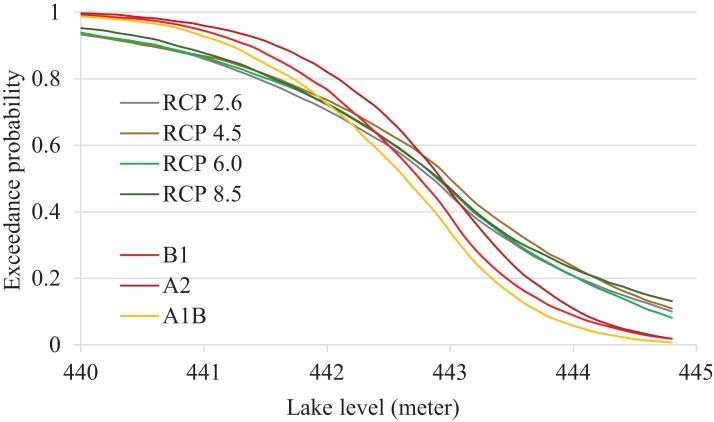
Exceedance probabilities of Devils Lake under different CMIP-5 and CMIP-3 emission scenarios. Note that 444.4 m above mean sea level is the lake spillover level.

Warmer temperature and higher precipitation under CMIP-5 climate projections led to increased surface runoff (SQ) by 10.8%, compared to CMIP-3 simulations; an increase only partially compensated by a temperature-driven increase in ET of less than 1%. The overall change in water balance was higher under CMIP-5 simulations; amplified over multiple years, these differences led to higher probabilities of DL overspill to the Sheyenne River (Table 5). Notably, the CMIP-5 ensemble simulations projected significantly higher OP: compare the mean OP across all GCMs and RCPs of 37.8% (CMIP-5) with 12.8% under CMIP-3 ensemble (Fig. 4). We found uncertainty between the GCMs in terms of overspill risks with OP ranging from 0% to 100%. Some members of the CMIP-5 GCMs (BCC-CSM1.1m, IPSL-CM5A-LR, IPSL-CM5A-MR, MIROC-ESM, MIROC-ESM-CHEM) indicated 100% overspill probabilities while some others (CSIROMk360, GFDLESM2G) indicated as low as zero OP (Table 6). The higher overspill probabilities could be attributed to two factors observed in CMIP-5 climate projections: increased winter temperatures that could speed up snow melt and increased precipitation during April and May.

**Table 5:** Simulated mean evapotranspiration (ET), surface runoff (SQ) and Devils Lake overspill probability (OP) under future climate.

Climate	CMIP-5				CMIP-3			
	Scenario	ET (mm)	SQ (mm)	OP (%)	Scenario	ET (mm)	SQ (mm)	OP (%)
2020s	RCP2.6	485.7	22.41	40.6	–			
	RCP4.5	486.5	22.69	47.1	B1	481.8	21.30	17.7
	RCP6.0	482.0	22.21	40.9	A1B	483.1	20.65	11.7
	RCP8.5	487.5	23.43	44.4	A2	486.5	21.78	20.6
2050s	RCP2.6	489.1	20.96	33.5	–			
	RCP4.5	499.9	20.99	36.2	B1	490.8	19.30	10.9
	RCP6.0	487.9	19.26	24.4	A1B	495.0	17.69	8.3
	RCP8.5	504.2	22.20	35.6	A2	487.2	17.19	7.8

**Notes:**

SWAT simulated mean annual evapotranspiration, surface runoff and Devils Lake overspill probability under the downscaled CMIP-5 and CMIP-3 integrations for 2020s and 2050s.

The CMIP-5 and CMIP-3 scenarios are arranged in order of their approximate correspondence.

**Table 6:** Devils Lake overspill probability (%) under four representative concentration pathways (RCPs) for 2020s and 2050s CMIP-5 climate as projected by 17 global circulation models.

GCMs	Overspill probability (%)							
	2020s				2050s			
	RCPs							
	2.6	4.5	6.0	8.5	2.6	4.5	6.0	8.5
BCC-CSM1.1	10	35	20	15	20	10	15	0
BCC-CSM1.1(m)	95	95	90	30	100	100	90	100
CSIRO-Mk3.6.0	0	10	5	5	5	0	0	0
FIO-ESM	20	15	10	15	10	30	30	0
GFDL-CM3	50	0	10	50	0	10	5	5
GFDL-ESM2G	0	0	5	0	0	0	0	0
GFDL-ESM2M	0	30	0	90	10	0	15	0
GISS-E2-H	40	50	25	45	20	25	20	25
GISS-E2-R	70	75	60	90	70	30	25	55
HadGEM2-ES	45	80	90	90	25	35	10	100
IPSL-CM5A-LR	100	100	20	35	100	100	70	35
IPSL-CM5A-MR	30	100	25	100	20	100	45	100
MIROC5	10	90	40	55	75	10	20	40
MIROC-ESM	100	45	100	100	50	75	10	100
MIROC-ESM-CHEM	40	55	100	0	55	55	40	0
MRI-CGCM3	65	15	15	10	0	5	0	35
NorESM1-M	15	5	80	25	10	30	20	10

### Devils Lake overspill risks under flood mitigation scenarios

**Figure 5:**
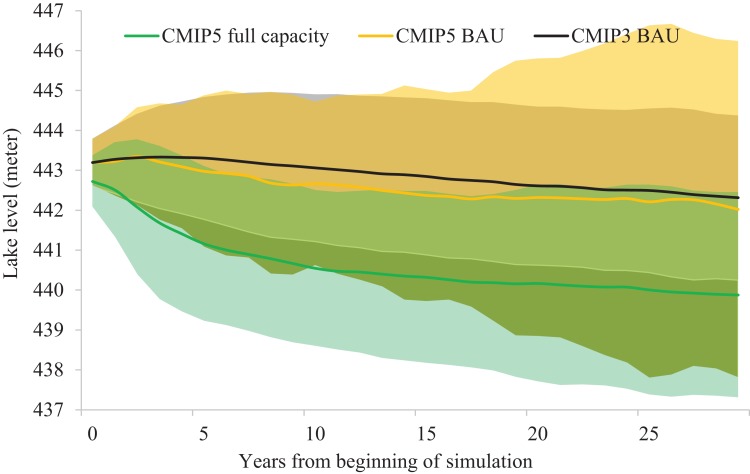
Simulated water level timeline visualizing model- and climate-related uncertainly. Green and orange solid lines represent the full outlet capacity and business-as-usual (BAU) simulations under the CMIP-5 RCP6.0 2020s climate. Black solid line represents the BAU simulations under the CMIP-3 A2 2020s climate, which is roughly equivalent to CMIP-5 RCP6.0. The two standard deviation wide shadow areas encompassing the solid lines represent visualized propagation of uncertainly related to differences in GCM output.

**Figure 6:**
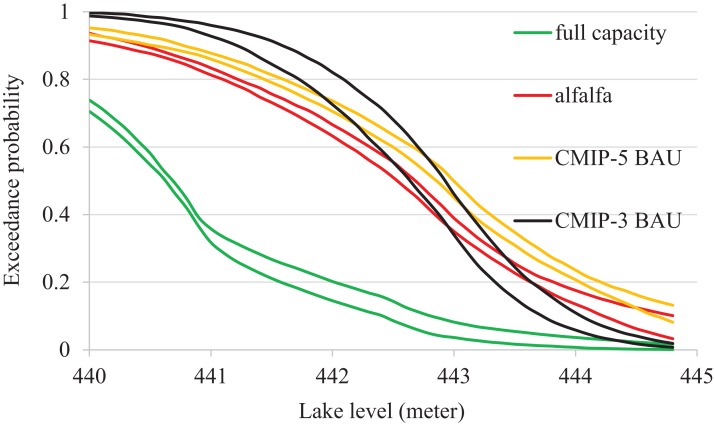
Exceedance probabilities of Devils Lake for different mitigation scenarios. Note that 444.4 m above mean sea level is the lake spillover level. Two lines reflect the uncertainty due to different emission scenarios (see Fig. 4 for comparison).

We found that none of the existing flood mitigation scenarios would prevent DL overspill under the CMIP-5 2020s climate condition. Running the outlets at full-capacity significantly reduced the overspill risks from 40.9–47.1% to 3.5–14.4%, which is still high compared to a negligible risk under the CMIP-3 integrations (Tables 5 and 7). Some members of the CMIP-5 GCMs indicated very high overspill probabilities; for example, MIROC-ESM (80%) and IPSL-CM5A-MR (55%). The model- and climate-related uncertainty are visualized in Fig. 5. In the Kharel, Zheng & Kirilenko (2016) land use management modeling study, the authors suggested that giving a $20/acre incentive to farmers for replacing row crops with alfalfa hay production which would reduce surface runoff and thereby reduce the upper basin streamflow contribution to the lake, and ultimately reduce the DL’s overspill risk. In this study using the CMIP-5 climate ensemble, it was shown that relying on incentivized alfalfa production alone led to the unacceptably high OP of approximately 30%. A combination of full-capacity outlet operation and providing incentives for replacement of crops with hay production is the most effective in mitigating DL flooding (Fig. 6). However, the risks may still be unacceptable given very high potential damage of downstream flooding, implying that more management alternatives may be necessary to protect vulnerable communities.

**Table 7:** Flood risks associated with three mitigation scenarios under the 2020s CMIP-5 and CMIP-3 climate.

Flood mitigation scenarios	CMIP-5		CMIP-3	
	Scenario	OP (%)	Scenario	OP (%)
Full-capacity outlet	RCP2.6	7.6	–	
	RCP4.5	7.6	B1	0.0
	RCP6.0	3.5	A1B	0.0
	RCP8.5	14.4	A2	0.0
Incentivized alfalfa	RCP2.6	30.6	–	
	RCP4.5	32.1	B1	8.6
	RCP6.0	29.1	A1B	5.0
	RCP8.5	30.9	A2	10.0
Full-capacity outlet + alfalfa	RCP2.6	4.1	NA	
	RCP4.5	5.3		
	RCP6.0	1.2		
	RCP8.5	11.5		

## Discussions

DL flooding is one of the most expensive, controversial, and complicated water resource management issues in the Northern Great Plains region. Managing and mitigating DL flooding has cost more than 1.5 billion US dollars, threatened the Boundary Waters Treaty between the US and Canada, motivated several court cases, and involved more than four dozen organizations and stakeholders. This multi-decadal pattern of DL flooding was attributed to abnormally high regional precipitation since the 1980s (Kharel & Kirilenko, 2015; Vecchia, 2011). Despite that, the currently implemented mitigation strategies take no account of future changes in climate, limiting their effectiveness. Previous studies of future DL catastrophic flood risks that took into account climate change found the lake OP to be 7.8–20.6% in the absence of mitigation management and no OP with operating water outlets (Kharel & Kirilenko, 2015; Kharel, Zheng & Kirilenko, 2016). This study found that the CMIP-5 climate projections for the decades 2020s and 2050s result in a significantly higher risks of DL overspill of 24.4–47.1%, which is twice as high as under the CMIP-3 projections. The elevated overspill risks are related to a projected increase in precipitation, especially in the months of April and May when lake elevation increases the most. Additionally, warmer spring temperature reduces snow accumulation and accelerates the melting of snowpack (Cooper, Nolin & Safeeq, 2016; Diffenbaugh, Scherer & Ashfaq, 2013) resulting in a flash of spring runoff into the lake.

For many parts of the world, GCMs generally are in agreement on future shifts of precipitation towards a wetter or a dryer pattern. However, for the region of our study, we found considerable variation in GCM projections. As the range of the future climate prediction window increases, those GCM variations contribute to amplification of hydrologic model uncertainty (Fig. 6) and increase predicted risks of the lake overspill. The last IPCC report lists three sources of these uncertainties: the inter-model differences in parameterization of physical processes; the uncertainty in future driving forces of climate; and the internal variability of climate (IPCC, 2012). The latter two components of uncertainty are impossible to reduce, but quantification of these uncertainties e.g., through employing multi-model ensembles should help in estimation of future climate-related risks.

A long-term goal of the DL management team is to lower the DL water level to 441.35 m (1.2 m below the current level) via outlets (North Dakota State Water Commission, 2017), but this plan does not specify particular mitigation strategy in case of an emergency overspill. Current flood management mainly relies on diverting flood water to the nearby Sheyenne River via two artificial outlets, which greatly reduce DL overspill risks under the current climate. However, hydrologic modeling using the CMIP-5 GCM ensemble showed that the likelihood of lake overspill would be high even when the outlets run at their combined capacity of 17.0 m^3^/s for six months. Even this type of flood management might not be attainable due to concerns on downstream flooding and water quality which have already been reported in downstream communities (Haley, 2014). Previous research found that running both outlets at their full-capacity would degrade the water quality of the Sheyenne River and also would violate the current North Dakota sulfate concentration water quality standard of 450 mg/l (Shabani, Zhang & Ell, 2017). Further, on multiple occasions the outlet operations were suspended due to mechanical failures or legal constraints; between 2012 and 2017, the average outlet release rate was only 10.1 m^3^/s (North Dakota State Water Commission, 2017, 2018).

After more than two decades of flooding, management conflicts, negotiations, and over a billion dollars in expenses, the outlet-based mitigation remains the single major solution. If future climate indeed elevates flood risks, the current outlets are likely to become inadequate and require increased capacity. However, the success of this outlet-based flood mitigation greatly depends on previously agreed conditions between the US and Canada in 2005 (Paris, 2008) mostly related to downstream water quality protection and flood prevention (International Red River Board, 2016). The existing water management related treaties and agreements, including the Boundary Waters Treaty and the US-Canada 2005 DL agreement (Paris, 2008), lack climate change impact consideration to cover the associated water quality and flood issues. This presents two management options. The first option is finding a way to relax current regulations on downstream water quality. Given that water-related problems in the downstream communities are reported even under the current operating schedule (North Dakota State Water Commission, 2017), realization of this option is highly unlikely. The second option is to actively pursue other alternatives such as sustainable land use management in the upper lake watershed. It would be attractive to explore restoration and protection of wetlands as a companion solution together with outlet operation to minimize the flooding risks due to climate change.

Given that full-capacity outlet operation is unrealistic, future climate projections indicate DL water overspill is even more likely than currently projected. Combining mechanical water removal with land management through replacement of croplands with hay production would significantly lower flood risks to 12% on average in our simulation, which is still high given the high potential damage of an overspill event. Restoration of wetlands within the watershed may further reduce flood risks as wetlands function as buffer and control flooding (Bullock & Acreman, 2003), and has been proposed in the “three-prong approach” to DL management (Devils Lake Basin Joint Water Resource Board, 2013). The DL watershed is part of the Prairie Pothole Region (PPR) in the northern Great Plains, which is characterized by thousands of wetlands with a “fill-and-spill” runoff system (Mekonnen et al., 2014). Roughly half of these wetlands have been drained for agricultural purpose in the region. Even though the protection and restoration of upper basin wetlands are considered as flood mitigation strategies, at the time of this study less than 1% of the watershed with less than 10% of the wetlands have been restored and protected (Devils Lake Basin Joint Water Resource Board, 2013). It could be due to a low benefit-to-cost ratio estimated at 0.2 to 1 (Gehringer & Hove, 1998), this project received minimal success. It was estimated that restoring the 374 km^2^ of historical wetlands in the DL watershed could reduce surface runoff by roughly 9% (West Consultants Inc., 2001).

Wetlands of the PPR have a huge range of hydro-topographic characteristics such as size, distribution, connectivity, snow and water accumulation, and drainage area and pattern (Chu, 2015), making modeling wetlands a difficult task. Accordingly, we excluded modeling of wetlands and potholes in this study due the extensive data and time needed for the effort, and therefore the impact of wetland buffering capacity in controlling flood was not fully accounted for in estimating the lake’s overspill risks. Additionally, during the model simulation period, we assumed no changes in the watershed land cover types. Vegetation cover, land use changes and associated parameters such as albedo and surface roughness are key to soil water distribution, streamflow and groundwater recharge (Acharya et al., 2017a, 2017b). However, previous studies speculated negligible impacts of groundwater recharge on the DL water level fluctuations. We recommend incorporating detailed modeling of land modifications, wetlands, and groundwater recharge in future research.

## Conclusion

This study estimated the potential flood risk in DL watershed in North Dakota under the CMIP-5 climate conditions. The current flood management based on the outlet operation seems adequate based on the earlier CMIP-3 climate projections. Management solutions to reduce the DL water levels based on land use change in the upper watershed appear effective if resource managers and policy makers consider crop incentives in their mitigation plans. However, the latest CMIP-5 GCM simulations indicate an increase in both precipitation and temperature in the region as compared to the CMIP-3 projections, leading to increased DL overspill risk as compared to the previous estimations. The wide range of climate projections among the CMIP-5 ensemble of models led to a large degree of uncertainty in terms of overspill risks, with OP ranging between 0% and 100%, with an average of 38% exceedance probability across the GCMs and RCPs. None of the flood mitigation strategies investigated in this study would eliminate the overspill risk. However, a combination of full-capacity outlet operation with sustainable land use in the upper watershed could significantly lower DL OP to just 5.5% on average. The study results could be viewed both as a warning sign and useful information to resource managers and stakeholders in the region to explore alternative solutions to mitigate the impact of climate change in the overall management of DL and downstream communities.

## Supplemental Information

10.7717/peerj.4711/supp-1 Supplemental Information 1 Hydrological model of Devils Lake watershed based on SWAT.

10.7717/peerj.4711/supp-2 Supplemental Information 2 CMIP-5 and CMIP-3 climate data for Devils Lake watershed.

10.7717/peerj.4711/supp-3 Supplemental Information 3 Simulated and observed daily streamflows at the seven USGS stations within the Devils Lake watershed.

10.7717/peerj.4711/supp-4 Supplemental Information 4 SWAT model parameter values.

10.7717/peerj.4711/supp-5 Supplemental Information 5 Values of surface runoff curve number (CN2) used in the model for different land use types in each sub-basin.

10.7717/peerj.4711/supp-6 Supplemental Information 6 Python scripts used to extract climate data and analyze simulation results.

## Additional Information and Declarations

### Data Availability

The following information was supplied regarding data availability: The raw data and python codes are provided as Supplemental Files. Kharel, Gehendra (2018): Devils Lake SWAT model output. figshare. Fileset. https://doi.org/10.6084/m9.figshare.6007643.v1.
