# Supplementary figures and images for: Comparing CMIP-3 and CMIP-5 climate projections on flooding estimation of Devils Lake of North Dakota, USA
Source: PeerJ. 2018 Apr 30;6:e4711. doi: 10.7717/peerj.4711 (PMC5933320; doi:10.7717/peerj.4711)

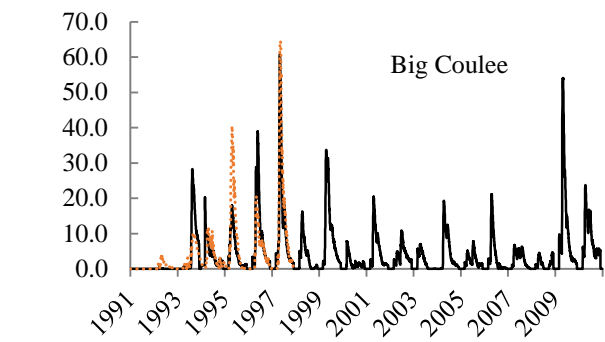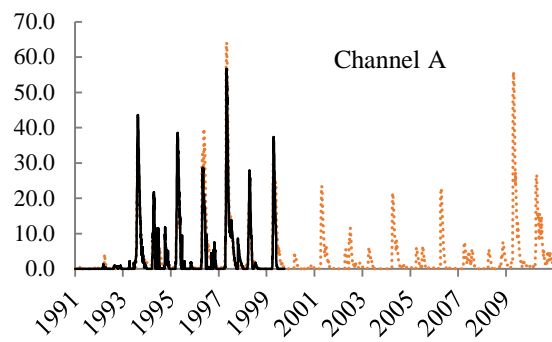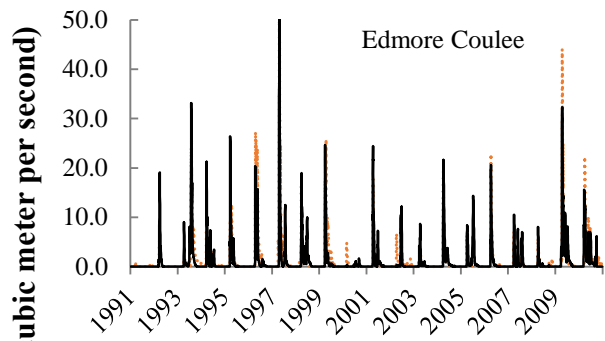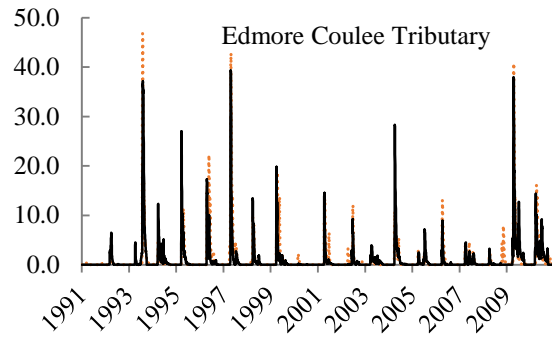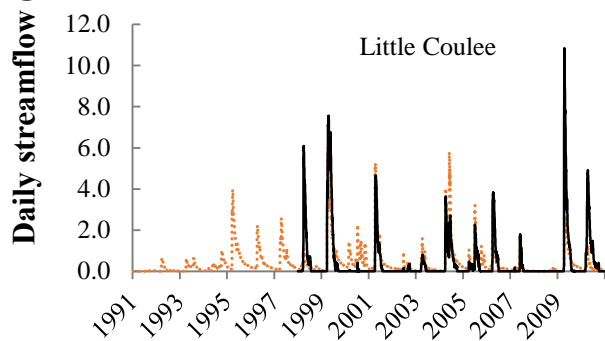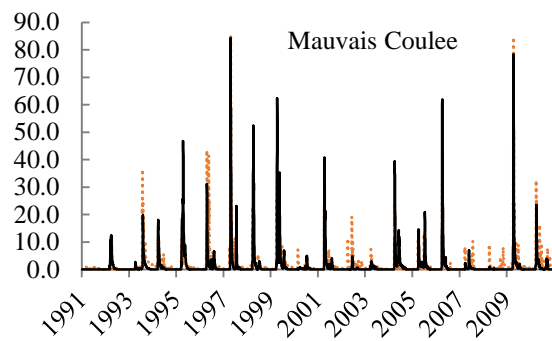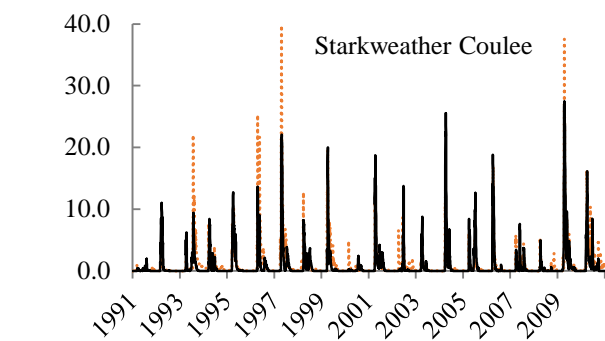

..... SWAT simulated  
—— USGS observed

Time

Supplement: Supplemental Information 3 [file peerj-06-4711-s003.pdf]
