# Supplementary material for: Comparing CMIP-3 and CMIP-5 climate projections on flooding estimation of Devils Lake of North Dakota, USA
Source: PeerJ. 2018 Apr 30;6:e4711. doi: 10.7717/peerj.4711 (PMC5933320; doi:10.7717/peerj.4711)
Supplement: Supplemental Information 4 [file peerj-06-4711-s004.docx]

| **Sub-basin** | **ESCO** | **EPCO** | **SURLAG** | **CH_N2** | **ALPHA_BNK** | **CH_N1** |
| --- | --- | --- | --- | --- | --- | --- |
| **1** | 0.16 | 0.92 | 0.9 | 0.02 | 0.79 | 0.08 |
| **2** | 0.8 | 0.85 | 0.49 | 0.09 | 0.88 | 0.032 |
| **3** | 0.47 | 0.6 | 0.46 | 0.06 | 0.57 | 0.016 |
| **4** | 0.47 | 0.4 | 0.55 | 0.02 | 0.35 | 0.05 |
| **5** | 0.09 | 0.5 | 0.9 | 0.075 | 0.25 | 0.014 |
| **6** | 0.48 | 0.87 | 0.19 | 0.058 | 0.44 | 0.041 |
| **7** | 0.65 | 1 | 0.12 | 0.1 | 0.71 | 0.05 |
| **8** | 0.1 | 1 | 0.1 | 0.054 | 0.1 | 0.078 |
| **9** | 0.48 | 0.87 | 0.19 | 0.058 | 0.44 | 0.041 |
| **10** | 0.1 | 1 | 0.1 | 0.054 | 0.1 | 0.078 |
| **11** | 0.75 | 0.75 | 0.9 | 0.055 | 0.46 | 0.043 |
| **12** | 0.75 | 0.75 | 0.9 | 0.055 | 0.46 | 0.043 |
