# Supplementary material for: Comparing CMIP-3 and CMIP-5 climate projections on flooding estimation of Devils Lake of North Dakota, USA
Source: PeerJ. 2018 Apr 30;6:e4711. doi: 10.7717/peerj.4711 (PMC5933320; doi:10.7717/peerj.4711)
Supplement: Supplemental Information 5 [file peerj-06-4711-s005.docx]

| **Sub-basin** | **Agriculture** | | | | | | | | **Non-agriculture** | | | | |
| --- | --- | --- | --- | --- | --- | --- | --- | --- | --- | --- | --- | --- | --- |
|  | **Alfalfa** | **Barley** | **Canola** | **Corn** | **Beans** | **Soybean** | **Sunflower** | **Wheat** | **Grassland** | **Forest** | **Wetlands** | **Developed** | **Water** |
| **1** | 67.0 | 75.3 | 77.2 | 77.2 | 79.1 | 79.1 | 77.2 | 75.3 | 67.0 | 67.9 | 73.5 | 67.0 | 92.0 |
| **2** | 54.9 | 67.9 | 71.6 | 71.6 | 72.5 | 72.5 | 77.2 | 67.9 | 54.9 | 67.9 | 64.2 | 54.9 | 92.0 |
| **3** | 67.0 | 75.3 | 77.2 | 77.2 | 72.5 | 79.1 | 77.2 | 75.3 | 67.0 | 67.9 | 73.5 | 67.0 | 92.0 |
| **4** | 54.9 | 75.3 | 77.2 | 77.2 | 79.1 | 79.1 | 77.2 | 75.3 | 54.9 | 67.9 | 64.2 | 67.0 | 92.0 |
| **5** | 54.9 | 75.3 | 71.6 | 77.2 | 79.1 | 79.1 | 71.6 | 75.3 | 54.9 | 55.8 | 64.2 | 54.9 | 92.0 |
| **6** | 67.0 | 75.3 | 77.2 | 77.2 | 79.1 | 79.1 | 77.2 | 75.3 | 67.0 | 67.9 | 73.5 | 67.0 | 92.0 |
| **7** | 67.0 | 67.9 | 71.6 | 77.2 | 72.5 | 72.5 | 71.6 | 67.9 | 54.9 | 55.8 | 73.5 | 54.9 | 92.0 |
| **8** | 67.0 | 75.3 | 77.2 | 77.2 | 79.1 | 79.1 | 77.2 | 75.3 | 67.0 | 73.5 | 64.2 | 67.0 | 92.0 |
| **9** | 54.9 | 75.3 | 77.2 | 71.6 | 72.5 | 72.5 | 71.6 | 67.9 | 54.9 | 55.8 | 64.2 | 54.9 | 92.0 |
| **10** | 54.9 | 67.9 | 77.2 | 77.2 | 79.1 | 79.1 | 77.2 | 75.3 | 54.9 | 67.9 | 73.5 | 67.0 | 92.0 |
| **11** | 58.7 | 72.6 | 76.6 | 76.6 | 77.6 | 77.6 | 76.6 | 72.6 | 58.7 | 72.6 | 78.6 | 58.7 | 92.0 |
| **12** | 58.7 | 72.6 | 76.6 | 76.6 | 77.6 | 77.6 | 76.6 | 72.6 | 58.7 | 59.7 | 68.7 | 58.7 | 92.0 |
